# Use of PEG-asparaginase in monomorphic epitheliotropic intestinal T-cell lymphoma, a disease with diagnostic and therapeutic challenges

**DOI:** 10.3332/ecancer.2017.771

**Published:** 2017-09-27

**Authors:** Cesar Gentille, Qian Qin, Andreia Barbieri, Pingali Sai Ravi, Swaminathan Iyer

**Affiliations:** 1 Department of Internal Medicine, Houston Methodist Hospital, Houston, Texas 77030, USA; 2 Department of Pathology, Houston Methodist Hospital, Houston, Texas 77030, USA; 3 Houston Methodist Cancer Center, Houston Methodist Hospital, Houston, Texas 77030, USA

**Keywords:** MEITL, EATL, asparaginase

## Abstract

Monomorphic epitheliotropic intestinal T-cell lymphoma (MEITL), previously known as enteropathy associated T-cell lymphoma (EATL) type II, is a rare haematological malignancy with a difficult and delayed diagnosis. Symptoms can include abdominal pain, weight loss, and chronic diarrhoea. However, most patients are only diagnosed after complications, such as perforation or obstruction, have developed. There is no standard treatment for MEITL; most accepted regimens consist of surgical resection and anthracycline-based chemotherapy. Prognosis is poor with an approximate survival of less than a year. Even though other therapies, such as autologous stem cell transplant, has shown promising results, not all patients can tolerate this course of treatment especially if they are elderly, have several comorbidities or are malnourished. Innovative therapies that improve survival and can be used as an alternative for more intensive treatment are needed. We report the use of PEG-asparaginase along with conventional anthracycline therapy in a 70-year-old woman diagnosed with MEITL, who went into remission and survived for more than one year before succumbing to relapsed disease.

## Background

Enteropathy associated T-cell lymphoma (EATL) type II, now referred to as monomorphic epitheliotropic intestinal T-cell lymphoma (MEITL), is a rare primary extranodal T-cell lymphoma arising from the intraepithelial lymphocytes of the small intestine and presenting with non-specific abdominal pain weight loss and diarrhoea [1, 2]. Most patients are diagnosed due to complications such as perforation, bleeding, or obstruction [1, 4]. Celiac disease is strongly associated with EATL type I and diagnosed at an earlier stage compared to MEITL where this association is lacking [1, 4, 5]. There is no standard therapy for MEITL, and patients often receive combined modality treatments that include surgery, chemotherapy, and radiotherapy. The most widely used regimen appears to be a systemic anthracycline-containing chemotherapy with primary surgical resection [1, 3–6]. CHOP is the most frequently used chemotherapy with an anticipated 5-year survival rate of ~20% [7–9]. The role of surgery besides being initially diagnostic is limited to tumour debulking and resection of masses with high risk of obstruction or perforation before initiating chemotherapy [3, 4]. Combination therapy with surgery and chemotherapy is superior to patients treated with surgery alone [8]. However, relapse rates using these regimens have been reported to be as high as 80% with a similar mortality rate around 80–85% [4, 6, 7, 10]. New therapies are being researched due to the poor outcomes with the conventional regimen.

Here, we describe a unique case of MEITL where a patient with significant pre-existing comorbidities tolerated infusion anthracycline therapy with PEG-asparaginase after surgical resection of the tumour.

### Case presentation

A seventy-year-old Hispanic lady with a history of diabetes mellitus type 2, cerebrovascular accident with residual right hemiparesis and peripheral vascular disease presented with intermittent lower abdominal pain, nausea, vomiting and diarrhoea for 14 months. She had an unintentional weight loss of 50 lbs without any history of fever, rigors, night sweats or chills.

Abdominal examination revealed hyperactive bowel sounds and diffuse tenderness, without any signs of peritonitis. Laboratory studies were significant for mild leukocytosis (WBC 12.8 × 10^9^/L) and anaemia (Hb 9.9 gm/dL), no electrolyte abnormalities or elevation of lactic acid was noted. Hypoalbuminaemia (2.8 mg/dL) was also seen.

**Figure 1.**
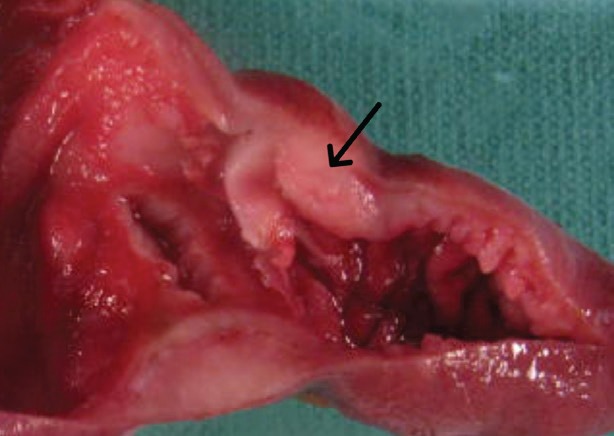
Segment of small intestine with prominent wall thickening and stricture formation (arrow).

**Figure 2.**
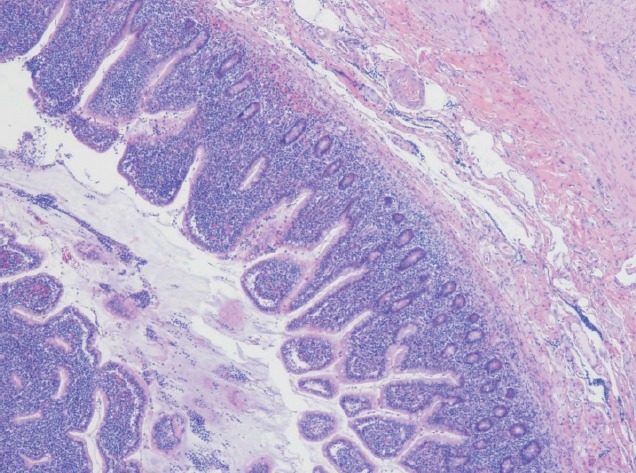
Small intestinal mucosa with villous blunting and florid infiltration of the lamina propria, intestinal crypts, and surface epithelium by small- to medium-sized monomorphic lymphoma cells.

**Figure 3.**
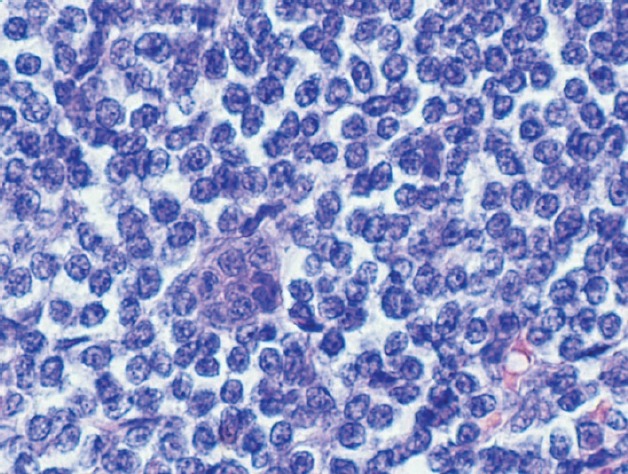
Monomorphic small- to medium-sized lymphoma cells with round nuclei, slightly irregular nuclear contour, vesicular chromatin, and rim of clear cytoplasm.

Computerised tomographic scan of abdomen and pelvis showed mechanical obstruction at the jejunoileal junction with marked distention of the small bowel, suspicious for carcinoma. A large tumour measuring 10 cm in length and 8.5 cm in diameter causing almost complete obstruction was resected. The tumour had a multinodular appearance with a prominent wall thickening and a soft, homogenous cut surface with stricture formation (Figure 1). On microscopic examination, it was composed of monomorphic small lymphocytes positive for CD3, CD8, CD56 which was consistent with monomorphic epitheliotropic intestinal T-cell lymphoma (Figure 2, 3). The tumour was also positive for T-cell receptor gamma and negative for CD4, CD20, and CD30.

Staging PET scan showed increased uptake in the hepatic flexure and some mucosal thickening. Colonoscopy revealed colonic ulcers and strictures in the ascending colon. The patient then went on to receive a right hemicolectomy with a subsequent histopathological analysis also consistent with MEITL. Bone marrow aspiration and biopsy did not show any evidence of disease.

The patient received four cycles of etoposide, prednisone, vincristine, cyclophosphamide, and doxorubicin (EPOCH) chemotherapy. However, a follow-up PET scan showed persistent uptake in the ascending colon. PEG-asparaginase was added to the 5th EPOCH cycle, and then, it was given as a single therapy monthly. Two months later, a new PET scan showed nearly complete resolution of uptake. Treatment regimen with PEG-asparaginase was continued with follow-up PET scans (3 and 9 months after resolution) not showing any evidence of viable lymphoma.

### Follow-up

Unfortunately, 15 months after her initial diagnosis, the patient developed abdominal pain and diarrhoea. A PET scan showed marked uptake in a segment of abnormal appearing bowel in the right ascending colon and distal descending colon similar to her first PET scan. Colonoscopy showed ulcers that were positive for MEITL on histological examination. She was started on EPOCH. However, she developed a small bowel obstruction soon after and the first cycle could not be completed. After recovering with medical management and temporary supportive care with total parenteral nutrition, she was taken to the operation room for laparotomy with lysis of adhesions and small bowel resection for tumour debulking. Despite having an apparent uneventful post-operative course and receiving one cycle of EPOCH and one dose of PEG-asparaginase, she had another episode of bowel obstruction 2 months later. Laparotomy and lysis of adhesions was again performed with microscopic examination still positive for MEITL.

She was readmitted two months later due to worsening lower quadrant abdominal pain. CT abdomen and pelvis showed numerous loops of dilated small bowel with marked thickening and free intraperitoneal air consistent with bowel perforation. The patient was deemed not a surgical candidate because of worsening performace status, comorbidities after multiple surgeries and overall poor prognosis. The family opted for hospice and comfort measures; the patient passed away shortly after.

## Discussion/literature review

EATL is an extranodal T-cell lymphoma that comprises 10–25% of all primary lymphomas of the small intestine. It was initially classified in two types, classic EATL or type I, was strongly associated with celiac disease and considered the most common form while type II comprised only 10–20% of all EATL and did not have an association with celiac disease [4, 11] However, given their distinct features, the World Health Organisation now distinguishes them as separate conditions. EATL type I has been designated as enteropathy associated T-cell lymphoma and type II as monomorphic epitheliotropic intestinal T-cell lymphoma (MEITL) [2].

The estimated annual incidence of EATL (including type I and MEITL) in western countries is about 0.5 to 1 per 1 million people [3, 6, 12, 13]. A review of the surveillance, epidemiology and end results database from 1973 to 2008 showed a progressive increase in the annual age-adjusted incidence in the United States, from 0.006 per 100,000 people to 0.024 per 100,000 people. The incidence increased with age for both sexes, although males had a higher incidence than females [14]. Notably, EATL type I appears to be more common in these regions (The US and Europe), while MEITL is predominant in Asia [15]. Both have been reported in the Hispanic population, but the actual incidence in this group is undetermined [13].

Clinical manifestations can be non-specific including a change in bowel habits, chronic diarrhoea, weight loss, and chronic abdominal pain [4, 6, 13]. Complications, such as small bowel obstruction, hemorrhage, and jejunal perforation, can happen easily and account for approximately half of the initial presentations [1, 3, 16, 17]. B-symptoms (fever, night sweats, fatigue), on the other hand, are uncommon [1]. Other more rarely reported manifestations include skin lesions due to cutaneous infiltration, ascites, seizures and altered mental status, peripheral nerve infiltration, obstructive jaundice, bilateral ovarian masses, renal failure due to kidney infiltration, pleural mass, infiltrative cardiomyopathy, eosinophilia, autoimmune hemolytic anaemia and hemophagocytic lymphohistiocytosis (HLH) [18–31].

Diagnosis of MEITL is challenging considering that patients do not usually have history of celiac disease and exhibit non-specific symptoms. Patients may initially present with obstruction or perforation that leads to many cases being diagnosed during laparotomy [4, 6, 7, 12]. The tumours are multifocal, forming ulcers, nodules, plaques, strictures, or large masses; they are mostly located in the jejunum or ileum, followed by stomach, colon, and rectum [1, 13, 32]. Histopathology examination and immunophenotyping are essential for diagnosis. Imaging modalities that can help with diagnosis and evaluate the extent of disease include PET scan, MR enteroclysis, and endoscopy [1, 3, 4, 33]. Dissemination is commonly found and may involve mesenteric lymph nodes, liver, spleen, lung, and skin; however, bone marrow compromise is unusual [4, 32].

On histopathological analysis, findings include a transmural infiltrate of monomorphic cells with obliteration of the normal intestinal architecture. The intraepithelial lymphocytes are usually CD3+, CD4+, CD8+, CD56+, and TCRβ+ [10, 32]. In contrast, type-I EATL has a polymorphic cellular appearance and is characterised by intraepithelial lymphocytes that are usually CD3+, CD5-, CD7+, CD8-, CD4-, CD56-, TCRβ + with the presence of cytotoxic markers (TIA-1, granzyme, and perforin) [4, 6, 32].

The prognosis is poor, with an approximate estimated median survival of 7–10 months [1, 7–9]. There is no standardised treatment for MEITL and regimens, such as chemotherapy, surgery, and radiotherapy, have shown poor overall outcomes. Of note, most of the data evaluating the effectiveness of treatment regimens on MEITL patients originates from retrospective single institution cohorts and subgroup analysis of intestinal non-Hodgkin lymphoma patient studies (done using the previous WHO EATL classification). Other therapies that have been investigated include high-dose chemotherapy followed by stem cell transplant (SCT). Analysis from retrospective, prospective and a peripheral T-cell lymphoma trial (EATL subgroup) reported an improvement with SCT in 5-year overall survival with a rate of 48–60% and in 5-year PFS ranging up to 38–52% [8, 11]. Similar results were found in the largest cohort of these studies (44 patients) with a 4-year PFS of 54% and a 4-year overall survival of 59% [34]. Case report and case series prior to the studies mentioned earlier, had shown contradictory outcomes with some patients achieving total remission after transplant while others relapsed and eventually died from disease progression [35–38].

A retrospective study of patients treated with resection, chemotherapy and autologous SCT had a higher 1-year and 5-year overall survival (100%, 33%) compared to one-year survival (73%) and five-year survival (14%) without autologous SCT [39]. These results were consistent with data reported by Sieniawski *et al*, where they compared outcomes after transplant with historical data from a previous cohort finding a significant difference between high-dose chemotherapy followed by SCT and standard-dose chemotherapy with or without surgical resection (5-year OS of 60% vs. 22% and 5-year PFS of 52% vs. 22%).^8^

Overall, a considerable improvement in survival after SCT has been seen; however, younger patients with few comorbidities who can tolerate the high-dose chemotherapy will probably benefit more than any other population. This therapeutic option needs to be considered cautiously, as many of them present with poor performance status and malnutrition, which may worsen tolerance to chemotherapy or impair them to finalise treatment [1, 6, 40].

Other alternative therapies have been tried in EATL and MEITL patients with favourable outcomes, including the adjuvant use of alemtuzumab and single use of brentuximab and romidepsin [1, 4, 12, 13]. Adjuvant PEG-asparaginase was used in the case of our patient, which has been scarcely reported in the literature.

L-Asparaginase is an enzyme also known as aminohydrolase that can break down L-asparagine into aspartate and ammonia [41]. Normal and leukaemic cells require asparagine as a substrate for cell growth. Normal cells can synthesise it by themselves. However, leukaemia and lymphoma cells lack the key enzyme used for L-asparagine synthesis. The elimination of the asparagine obtained from the diet will lead to the starvation and death of the cancer cells. Due to this antineoplastic action, asparaginase has been used as part of therapeutic regimens for haematological malignancies such as acute lymphoblastic leukaemia and Hodgkin’s lymphoma. Conjugation with polyethylene glycol (PEG-asparaginase) can improve the enzyme’s bioavailability and reduce the immunological response against asparaginase [41, 42].

The role of L-asparaginase in MEITL is not clearly defined. However, its use has been reported in a multicentre retrospective study evaluating the treatment outcomes of EATL in 38 patients from Asiatic regions. In this cohort, no apparent difference was found in the overall response rate when L-asparaginase was compared to the anthracycline-based regimens (ORR: 42% vs. 50%). Nevertheless, only 5 of 38 patients received this drug [15]. Of note, these five patients had an initial diagnosis of gastrointestinal NK/T-cell lymphoma but had overlapping features of MEITL [43]. Research done in relapsed and refractory NK/T-cell lymphoma has shown good outcomes after using L-asparaginase with a complete remission rate as high as 88% [44]. Furthermore, guidelines produced by the Italian Society of Hematology and its affiliate societies (Italian Society of Experimental Hematology and Italian Group for Bone Marrow Transplantation) recommend its use in cases of extranodal peripheral T-cell lymphoma with systemic disease [45].^4^ As mentioned earlier, some studies report similarities between EATL and extranodal NK/T-cell lymphoma, particularly in MEITL where both can share a gamma delta T-cell origin [46].

Due to our patient’s comorbidities and complications, the patient received anthracycline-based chemotherapy and PEG-asparaginase. We continued PEG-asparaginase monthly to control her disease, and it remitted for one year, more than the expected median survival. However, she was unable to repeat this treatment after her relapse due to her further complications. Based on our experience, we believe that PEG-asparaginase likely had a role in her initial remission and overall disease control.

## Conclusion

MEITL is rare, and diagnosis is challenging, with patients presenting for examination when symptoms have become too severe, chronic or when a complication has developed. Prognosis is poor even with surgery and chemotherapy; however, other therapies, such as transplant, have shown an improvement in survival. More studies looking for innovative regimens that improve survival are needed, especially as an alternative for patients who cannot tolerate high-dose chemotherapy and transplant. PEG-asparaginase can be a promising alternative considering its particular antineoplastic action and effectiveness in other related lymphomas such as NK/T-cell lymphoma.

## References

[1] Di Sabatino A, Biagi F, Gobbi PG (2012). How I treat enteropathy-associated T-cell lymphoma. Blood.

[2] Swerdlow SH, Campo E, Pileri SA (2016). The 2016 revision of the world health organization classification of lymphoid neoplasms. Blood.

[3] Yang Y, Batth SS, Chen M (2012). Enteropathy-associated T cell lymphoma presenting with acute abdominal syndrome: a case report and review of literature. J Gastrointest Surg.

[4] Ferreri AJ, Zinzani PL, Govi S (2011). Enteropathy-associated T-cell lymphoma. Crit Rev Oncol Hematol.

[5] Honemann D, Prince HM, Hicks RJ (2005). Enteropathy-associated T-cell lymphoma without a prior diagnosis of coeliac disease: Diagnostic dilemmas and management options. Ann Hematol.

[6] Sieniawski MK, Lennard AL (2011). Enteropathy-associated T-cell lymphoma: epidemiology, clinical features, and current treatment strategies. Curr Hematol Malig Rep.

[7] Gale J, Simmonds PD, Mead GM (2000). Enteropathy-type intestinal T-cell lymphoma: clinical features and treatment of 31 patients in a single center. J Clin Oncol.

[8] Sieniawski M, Angamuthu N, Boyd K (2010). Evaluation of enteropathy-associated T-cell lymphoma comparing standard therapies with a novel regimen including autologous stem cell transplantation. Blood.

[9] Delabie J, Holte H, Vose JM (2011). Enteropathy-associated T-cell lymphoma: clinical and histological findings from the international peripheral T-cell lymphoma project. Blood.

[10] van de Water JM, Cillessen SA, Visser OJ Enteropathy associated T-cell lymphoma and its precursor lesions. Best Pract Res Clin Gastroenterol.

[11] d’Amore F, Relander T, Lauritzsen GF (2012). Up-front autologous stem-cell transplantation in peripheral T-cell lymphoma: NLG-T-01. J Clin Oncol.

[12] Zhang JC, Wang Y, Wang XF (2016). Type I enteropathy-associated T-cell lymphoma in the colon of a 29-year-old patient and a brief literature review. Onco Targets Ther.

[13] Grigg-Gutierrez NM, Estremera-Marcial R, Caceres WW (2015). Primary enteropathy-associated T-cell lymphoma type 2: an emerging entity?. Cancer Control.

[14] Sharaiha RZ, Lebwohl B, Reimers L (2012). Increasing incidence of enteropathy-associated T-cell lymphoma in the united states, 1973–2008. Cancer.

[15] Tse E, Gill H, Loong F (2012). Type II enteropathy-associated T-cell lymphoma: a multicenter analysis from the Asia lymphoma study group. Am J Hematol.

[16] Kinaci E, Gunes ME, Huq GE (2013). An unusual presentation of EATL type 1: Emergency surgery due to life-threatening gastrointestinal bleeding. Int J Surg Case Rep.

[17] Pun AH, Kasmeridis H, Rieger N (2014). Enteropathy associated T-cell lymphoma presenting with multiple episodes of small bowel haemorrhage and perforation. J Surg Case Rep.

[18] Varghese D, Haseer Koya H, Cherian SV (2013). Hemophagocytic lymphohistiocytosis: an uncommon presentation of enteropathy-associated T-cell lymphoma. J Clin Oncol.

[19] Webster A, Crea P, Bamford MW (2016). Enteropathy-associated T-cell lymphoma presenting as cutaneous deposits. Br J Haematol.

[20] Nishimura M, Tomo K (2011). A case of enteropathy-associated T-cell lymphoma: diagnosis by flow cytometric immunophenotyping and genome analysis using ascitic fluid. Int J Clin Oncol.

[21] Berman EL, Zauber NP, Rickert RR (1998). Enteropathy-associated T cell lymphoma with brain involvement. J Clin Gastroenterol.

[22] Gobbi C, Buess M, Probst A (2003). Enteropathy-associated T-cell lymphoma with initial manifestation in the CNS. Neurology.

[23] Yap YS, Cummins A, Blumbergs P (2005). Lymphomatous infiltration of the peripheral nervous system in enteropathy-associated T-cell lymphoma. Med J Aust.

[24] Buess M, Steuerwald M, Wegmann W (2004). Obstructive jaundice caused by enteropathy-associated T-cell lymphoma in a patient with celiac sprue. J Gastroenterol.

[25] Jacob PM, Nair RA, Mehta J (2014). Enteropathy associated T cell lymphoma-monomorphic variant, presenting as bilateral ovarian masses. Indian J Pathol Microbiol.

[26] Wali GN, Tyrrell HE, Collins GP (2015). A rare but potentially fatal cause of diarrhoea and weight loss: enteropathy-associated T-cell lymphoma. BMJ Case Rep.

[27] Doyel GJ, Rose JD, Kesteven PJ (1993). Pleural lymphoma in a patient presenting with malabsorption: An illustration of the clinicopathological behaviour in a case of enteropathy associated T cell lymphoma. Gut.

[28] Hovenga S, de Graaf H, Joosten P (2003). Enteropathy-associated T-cell lymphoma presenting with eosinophilia. Neth J Med.

[29] Wu PH, Chu KE, Lin YM (2015). T-cell lymphomas presenting as colon ulcers and eosinophilia. Case Rep Gastroenterol.

[30] Kato A, Takiuchi Y, Aoki K (2011). Enteropathy-associated T-cell lymphoma type II complicated by autoimmune hemolytic anemia. J Clin Exp Hematop.

[31] Prichard D, Jamal S, Fortune A (2010). Enteropathy associated T-cell lymphoma resulting in restrictive cardiomyopathy and mimicking myocardial infarction. BMJ Case Rep.

[32] Arps DP, Smith LB (2013). Classic versus type II enteropathy-associated T-cell lymphoma: diagnostic considerations. Arch Pathol Lab Med.

[33] Hong YS, Woo YS, Park G (2016). Endoscopic findings of enteropathy-associated T-cell lymphoma type II: a case series. Gut Liver.

[34] Jantunen E, Boumendil A, Finel H (2013). Autologous stem cell transplantation for enteropathy-associated T-cell lymphoma: a retrospective study by the EBMT. Blood.

[35] Al-Toma A, Verbeek WH, Visser OJ (2007). Disappointing outcome of autologous stem cell transplantation for enteropathy-associated T-cell lymphoma. Dig Liver Dis.

[36] Bishton MJ, Haynes AP (2007). Combination chemotherapy followed by autologous stem cell transplant for enteropathy-associated T cell lymphoma. Br J Haematol.

[37] Rongey C, Micallef I, Smyrk T (2006). Successful treatment of enteropathy-associated T cell lymphoma with autologous stem cell transplant. Dig Dis Sci.

[38] Jantunen E, Juvonen E, Wiklund T (2003). High-dose therapy supported by autologous stem cell transplantation in patients with enteropathy-associated T-cell lymphoma. Leuk Lymphoma.

[39] Nijeboer P, de Baaij LR, Visser O (2015). Treatment response in enteropathy associated T-cell lymphoma; survival in a large multicenter cohort. Am J Hematol.

[40] Ikebe T, Miyazaki Y, Abe Y (2010). Successful treatment of refractory enteropathy-associated T-cell lymphoma using high-dose chemotherapy and autologous stem cell transplantation. Intern Med.

[41] Batool T, Makky EA, Jalal M (2016). A comprehensive review on L-asparaginase and its applications. Appl Biochem Biotechnol.

[42] Cachumba JJ, Antunes FA, Peres GF (2016). Current applications and different approaches for microbial l-asparaginase production. Braz J Microbiol.

[43] Yamaguchi M, Kwong YL, Kim WS (2011). Phase II study of SMILE chemotherapy for newly diagnosed stage IV, relapsed, or refractory extranodal natural killer (NK)/T-cell lymphoma, nasal type: the NK-cell tumor study group study. J Clin Oncol.

[44] Kwong YL, Kim WS, Lim ST (2012). SMILE for natural killer/T-cell lymphoma: Analysis of safety and efficacy from the asia lymphoma study group. Blood.

[45] Corradini P, Marchetti M, Barosi G (2014). SIE-SIES-GITMO guidelines for the management of adult peripheral T- and NK-cell lymphomas, excluding mature T-cell leukaemias. Ann Oncol.

[46] Pongpruttipan T, Sukpanichnant S, Assanasen T (2012). Extranodal NK/T-cell lymphoma, nasal type, includes cases of natural killer cell and alphabeta, gammadelta, and alphabeta/gammadelta T-cell origin: a comprehensive clinicopathologic and phenotypic study. Am J Surg Pathol.

